# A Disproportionate Burden of Care: Gender Differences in Mental Health, Health-Related Quality of Life, and Social Support in Mexican Multiple Sclerosis Caregivers

**DOI:** 10.1155/2015/283958

**Published:** 2015-10-11

**Authors:** Paul B. Perrin, Ivan Panyavin, Alejandra Morlett Paredes, Adriana Aguayo, Miguel Angel Macias, Brenda Rabago, Sandra J. Fulton Picot, Juan Carlos Arango-Lasprilla

**Affiliations:** ^1^Virginia Commonwealth University, Richmond, VA 23284, USA; ^2^University of Deusto, 48007 Bilbao, Spain; ^3^University of Guadalajara, 44100 Guadalajara, JAL, Mexico; ^4^Instituto Vocacional Enrique Diaz de Leon, 44100 Guadalajara, JAL, Mexico; ^5^University of Maryland-Baltimore, Baltimore, MD 21201, USA; ^6^IKERBASQUE, Basque Foundation for Science, 48007 Bilbao, Spain

## Abstract

*Background*. Multiple sclerosis (MS) rates in Latin America are increasing, and caregivers there experience reduced mental and physical health. Based on rigid gender roles in Latin America, women more often assume caregiving duties, yet the differential impact on women of these duties is unknown. *Methods*. This study examined gender differences in mental health (Patient Health Questionnaire-9, Satisfaction with Life Scale, Rosenberg Self-Esteem Scale, State-Trait Anxiety Inventory, and Zarit Burden Inventory), health-related quality of life (HRQOL; Short Form-36), and social support (Interpersonal Support Evaluation List-12) in 81 (66.7% women) Mexican MS caregivers. *Results*. As compared to men caregivers, women had lower mental health (*p* = 0.006), HRQOL (*p* < 0.001), and social support (*p* < 0.001). This was partially explained by women caregivers providing care for nearly twice as many hours/week as men (79.28 versus 48.48, *p* = 0.018) and for nearly three times as many months (66.31 versus 24.30, *p* = 0.002). *Conclusions*. Because gender roles in Latin America influence women to assume more substantial caregiving duties, MS caregiver interventions in Latin America—particularly for women caregivers—should address the influence of gender-role conformity on care and psychosocial functioning.

## 1. Introduction

Multiple sclerosis (MS) is a chronic, progressive, disabling disease which affects an estimated 2.3 million people worldwide [1]. In Latin America in particular, the focus of the current study, the number of individuals diagnosed with MS has been increasing over the past two decades [2, 3]. MS is characterized by autoimmune damage to the myelin sheaths around nerve cells [4]. MS manifests via deficits in the domains of physical (pain and impairments in visual, motor, vestibular, and somatosensory systems), cognitive (attention, processing speed, learning, memory, and executive functions), and emotional (depression and anxiety) functioning [5–8].

Due to the wide-ranging difficulties associated with MS, individuals often require care and support from informal caregivers, usually family members [9]. Caregivers often provide significant assistance with personal, medical, homemaking, mobility, and leisure tasks [10–14]. Providing care for an individual with MS has been associated with reduced caregiver mental health, physical health, and quality of life, as well as social and financial difficulties [9, 15–18]. Compared with caregivers of individuals with other neurological conditions, the issues faced by MS caregivers unfortunately have received relatively little research attention [19].

However, some studies have begun to examine gender differences in the provision and effects of MS caregiving, with inconsistent results. For instance, men spousal MS caregivers report having fewer total resources and lower perceived social support than women caregivers [20]. Additionally, Buchanan and colleagues [21] found that men caregivers had 71.1% greater odds of reporting higher burden than women caregivers. The same group's earlier findings [22] indicated that greater burden in men caregivers was associated with more hours of care per week, as well as with greater restriction of caregivers' ability to perform daily activities.

On the other hand, Knight et al. [23] reported that women spousal MS caregivers had higher levels of burden than husbands. Patti and colleagues [9] similarly found that women caregivers had higher psychological morbidity than men caregivers and that women gender was one of the chief predictors of lower quality of life in caregivers. Higher burden scores were interpreted to be related to higher levels of physical distress and exhaustion/tiredness experienced by women caregivers, while lower morbidity in men caregivers was attributed to less perceived psychological stress or better coping skills.

In Latin America, MS caregiver adjustment may be based on unique traditions, cultural norms, and societal beliefs. For instance, individuals in Latin America are generally collectivist and have strong familial ties and a sense of obligation to support immediate and extended family members who are sick [24]. Latino caregivers may be particularly vulnerable to “role engulfment” (i.e., basing one's identity on the assumed role of a primary caregiver) and consequently experience greater psychological morbidity and burden than caregivers from other cultural groups [25].

In addition to strong family values, cultural norms in Latin America also influence rigid gender-role expectations [26]. For example,* machismo*, an ethos of masculine behaviors expected of men in Latin America, is a good predictor of Latino men's health-related behaviors [27]. It might contribute to poor coping mechanisms (e.g., not reporting medical problems or asking for help) and lead to men being less knowledgeable about health-related issues as men see themselves as less vulnerable to disease than women [28]. Women in Latin America also face rigid gender roles by which they are expected to take care of the family, cook and clean, care for children, be submissive to and take orders from husbands, and restrict leisure activities and socialization outside home, which may predispose women to experiencing higher mental health problems [29–31].

To our knowledge, very few studies have been conducted examining MS caregiver experiences in Latin America and none examining gender differences in caregiver psychosocial functioning despite the unique cultural norms and beliefs in this region. Therefore, the purpose of the current study is to examine gender differences in mental health, health-related quality of life, and social support in a group of MS caregivers from Guadalajara, Mexico. It is hypothesized that women MS caregivers will experience lower scores on measures of these outcomes than men.

## 2. Materials and Methods

### 2.1. Participants

A purposive sample of 81 caregivers was recruited from the Mexican Foundation for Multiple Sclerosis and the Department of Neurosciences at the University of Guadalajara, Mexico, to participate in this cross-sectional study. Caregivers had to have provided care for a minimum of six months and be the primary caregiver of a person with MS. Exclusion criteria consisted of having a history of serious neurological, psychiatric, or learning disability. See Table 1 for participant demographics broken down by participant gender.

### 2.2. Measures

#### 2.2.1. Demographic Information

Demographic items assessed participants' gender, age, romantic partnership status, years of education, employment status, hours per week and total months spent caring for the MS patient, and caregiver relationship to the patient (parent, sister/brother, son/daughter, uncle, aunt, partner/spouse, or other relatives). All scales used in this study were previously published and validated Spanish versions that had already undergone extensive validation and were the most common indices of mental health and HRQOL administered in studies of caregivers of individuals with neurological conditions in Latin America and especially Mexico.

#### 2.2.2. Patient Health Questionnaire-9 (PHQ-9)

The PHQ-9 is a 9-item self-administered screening measure of depressive symptoms used in clinical and epidemiological studies [32].

#### 2.2.3. Satisfaction with Life Scale (SWLS)

The SWLS is a 5-item self-report measure of global life satisfaction [33]. The Spanish version of the SWLS has well-established psychometric properties [34, 35].

#### 2.2.4. Rosenberg Self-Esteem Scale (RSES)

The RSES is a 10-item measure of perceived self-worth [36, 37]. The RSES has well-established psychometric properties [38], including 53 countries, with many in Latin America [37].

#### 2.2.5. State-Trait Anxiety Inventory (STAI)

The STAI is a 40-item self-report measure of anxiety with two subscales [39]. Intensity of anxiety as an emotional state is measured by the S-Anxiety subscale. Anxiety as a personality trait and not just temporary responses to a situation is measured with the T-Anxiety scale [40].

#### 2.2.6. Zarit Burden Interview (ZBI)

The ZBI [41] is a 22-item self-report questionnaire that evaluates a caregiver's health condition, psychological well-being, financial situation, and social life in the context of the caregiver-patient relationship. The Spanish version of the ZBI has good internal reliability [42, 43].

#### 2.2.7. Interpersonal Support Evaluation List-Short Version (ISEL-12)

The ISEL-12 is a 12-item self-report instrument that assesses perceived social support according to participants' ratings of the availability of various types of social support [44] across three subscales: belonging, appraisal, and tangible.

#### 2.2.8. Short Form Health Status Survey (SF-36)

The SF-36 was used to assess health-related quality of life (HRQOL) across eight domains [physical functioning, role-physical (role limitations due to physical health problems), bodily pain, vitality, social functioning, role-emotional (role limitations due to emotional distress), mental health, and general health]. The Spanish version of the SF-36 has high reliability and validity [45].

### 2.3. Procedure

The Institutional Review Board (IRB) at the Mexican Foundation of Multiple Sclerosis reviewed and approved the study protocol prior to recruitment. Prospective participants for the study were recruited by the Mexican Foundation for Multiple Sclerosis and the Department of Neuroscience at the University of Guadalajara, Mexico. All participants reviewed and signed a consent form prior to the beginning of the study. Demographic, mental health, social support, and HRQOL information from caregivers were collected during a 40-minute face-to-face interview by a psychologist under the supervision of a member of the university teaching staff. No participant incentives were provided.

### 2.4. Statistical Analyses

Two multivariate analyses of variance (MANOVAs) compared women and men caregivers' mental health and social support (MANOVA 1) and HRQOL (MANOVA 2) with preset significance level of *p* < 0.05. In the first MANOVA, participant gender (women versus men) was the independent variable, and participants' total scores on each of the measures of satisfaction with life, depression, burden, self-esteem, anxiety, and social support were the dependent variables. In the second MANOVA, the independent variable was the same, but the dependent variables were participants' scores on the eight indices of HRQOL. Post hoc univariate analyses of variance (ANOVAs) were run to identify the locations of significant differences between women and men in each of the MANOVAs when the omnibus MANOVAs were statistically significant. Demographic characteristics (age, marital status, relationship to patient, education, working outside the home, and weekly and total time caregiving) of women and men participants were compared using* t*-tests for continuous variables and *χ* ^2^ tests for nominal variables. Two MANCOVAs were then run in the same manner as the first two MANOVAs but including as covariates any demographic variables that differed between women and men. MANOVAs and MANCOVAs were run first instead of proceeding immediately to ANOVAs and ANCOVAs in order to control for the potentially substantial family-wise error involved in the latter approach. As a result, ANOVAs and ANCOVAs were run only if the initial MANOVAs and MANCOVAs were statistically significant with a stringent cutoff of *α* = 0.05.

## 3. Results

### 3.1. MANOVA 1

The first MANOVA revealed a statistically significant effect for participant gender, Pillai's Trace = 0.259,* F*(9, 69) = 2.681, *p* = 0.01, and *η* ^2^ = 0.259. As a result, nine post hoc univariate analyses of variance (ANOVAs) were run to identify the location of the significant differences between women and men on the mental health and social support variables. In each of these ANOVAs, the independent variable was participant gender, and the dependent variables were each of the mental health and social support variables used in the omnibus MANOVA. The results of these ANOVAs appear in Table 2.

Across every index except satisfaction with life, women reported substantially worse functioning on the mental health and social support variables than men. The effect sizes [45] of two of the social support differences were large and one medium; one of the five mental health differences was a small effect, two were medium, and three were large effect sizes. As a result, this study's hypothesis that women caregivers would have poorer mental health and social support than men found extremely strong support.

### 3.2. MANOVA 2

The second MANOVA revealed a statistically significant effect for participant gender, Pillai's Trace = 0.316,* F*(8, 72) = 4.17, *p* < 0.001, and *η* ^2^ = 0.316. As a result, eight follow-up univariate analyses of variance (ANOVAs) were run to identify the location of the significant differences between woman and men on health-related quality of life. In each of these ANOVAs, the independent variable was participant gender, and the dependent variables were each of the HRQOL scores in the omnibus MANOVA. The results of these ANOVAs appear in Table 2.

Across every index except role-physical and role-emotional, women reported substantially lower HRQOL than men. The effect sizes [45] of the six statistically significant effects were all large. As a result, this study's hypothesis that women caregivers would have lower HRQOL than men had strong support.

### 3.3. Gender Differences in Demographics

The* t*-tests and *χ* ^2^ tests examining gender differences in participant demographics found differences in age, relationship to the individual with MS, education, work status outside the home, and hours per week and total months spent caregiving. See Table 1 for these means and standard deviations. Because of the significant gender differences in demographics, these variables were included as covariates in multivariate analyses of covariance (MANCOVAs) 3 and 4, except for relationship status to the individual with MS because categorical data with multiple categories cannot be included meaningfully in a MANCOVA.

### 3.4. MANCOVA 3

A third MANCOVA was run in the same manner as the first MANOVA for the mental health and social support variables with the addition of the following covariates: age, years of school, employment status, hours per week providing care, and months as a caregiver. The third MANCOVA revealed a statistically significant effect for participant gender, Pillai's Trace = 0.222,* F*(9, 64) = 2.06, *p* = 0.050, and *η* ^2^ = 0.222. As a result, nine follow-up univariate analyses of covariance (ANCOVAs) with the five covariates were run to identify the location of the significant differences between woman and men on the mental health and social support variables. In each of these ANCOVAs, the independent variable was participant gender, and the dependent variables were each of the total mental health and social support scores in the omnibus MANCOVA. The results of these ANCOVAs appear in Table 3.

Five out of the nine ANCOVAs were statistically significant in that women reported higher state and trait anxiety, lower self-esteem, and lower appraisal and tangible social support. The effect sizes [45] of the two social support differences were medium; two out of three mental health differences were medium effects; and the other difference was a large effect. As a result, this study's hypothesis that women would have poorer mental health and social support than men, even while controlling for demographic variables, was supported.

### 3.5. MANCOVA 4

The fourth MANCOVA did not reveal a statistically significant effect for participant gender, Pillai's Trace = 0.184,* F*(8, 67) = 1.89, *p* = 0.075, and *η* ^2^ = 0.184. Because this MANCOVA was not statistically significant, no follow-up ANCOVAs were run, and the study's hypothesis that women would have lower HRQOL than men, even while controlling for demographic variables, was not supported.

## 4. Discussion

The purpose of this study was to examine gender differences in mental health, social support, and HRQOL in Mexican MS caregivers. As compared to men caregivers, women caregivers had lower scores across the three sets of variables. When covarying for demographic variables that differed as a function of gender, the gender difference in HRQOL was no longer significant, but the differences in mental health and social support remained. This was the first study to examine and find gender differences in the psychosocial functioning of MS caregivers in Latin America. This is particularly notable given that MS rates in Latin America are increasing and caregivers there experience reduced mental and physical health, which may be influenced by rigid gender roles with women often assuming more substantial caregiving duties.

### 4.1. Gender Differences in Mental Health and Social Support

The finding that women reported lower scores on the mental health and social support variables than men (except satisfaction with life) is consistent with previous research in other global regions. In a sample of MS caregivers from New Zealand, Knight et al. [23] found that female spouses had higher burden than husbands and Patti and colleagues [9] similarly found that Italian female caregivers had higher levels of psychological morbidity than male caregivers. However, these findings were also discrepant from those in Good, Bower, and Einsporn's [20] study in which men spousal MS caregivers in the US had lower perceived social support than women caregivers and from Buchanan and colleagues' [21] finding in which men caregivers, also in the US, had higher burden than women caregivers.

Possible reasons for these robust significant differences found here may be related to differences in sample characteristics of Knight et al. [23] and Good et al. [20] and this study's sample and cultural perspectives on disability and caregiving. Both Knight et al. and Good et al. studied only MS spousal caregivers, and caregiver relationships to the MS patient may have a differential influence on mental health outcomes. More women were parents and more men were spouses and partners in the current study. In Mexican families, parents look to their adult children to provide them with care in old age and not the reverse [46]. Although women caregivers reported generally lower mental health than men, perhaps these differences were due also to the high self-sacrifice involved in fulfilling traditional nurturing and caregiving gender roles [29–31], and they did not report lower satisfaction with life, perhaps because fulfilling their gender role to provide care for a family member may have actually provided them with life satisfaction as they fulfilled a culturally prescribed role.

When age, years of school, employment status, hours per week providing care, and months as a caregiver were entered as covariates, the overall model still suggested that gender differences were present. However, in the follow-up ANCOVAs, several previously significant gender differences were no longer significant including depression, burden, and belonging social support. In previous studies, both employment and hours of caregiving have contributed significantly to caregiver burden and depression [19, 21, 22]. However, in this sample significantly more men worked, and women provided significantly more caregiving hours. Thus, controlling for these variables likely reduced their influences on the mental health and social support outcomes and pointed to a possible source of the gender difference on these variables.

Yet, differences still remained for appraisal and tangible social support, self-esteem, and state and trait anxiety. Cultural explanations of disability as either God's will or punishment for some previous actions of the parent have been described in Latin American cultures [47] and may account for the persistence of these results. Either belief could make appraisal and tangible social support for caregivers who are mothers less forthcoming and could place women caregivers of adult children at higher risk for lower mental health than the men caregivers of their spouses in this sample. Furthermore, awareness that these adult children with MS will be unavailable to provide care one day for these women caregivers in their old age could contribute to a lower self-esteem and higher levels of anxiety about their future.

### 4.2. Gender Differences in HRQOL

The finding that women reported lower HRQOL than men (except role limitations: physical and role limitations: emotional) is also generally consistent with previous research [48]. Studies by Rivera-Navarro and colleagues [49], as well as Patti and colleagues [9] and Giordano and colleagues [50], found that female gender was a major predictor of lower quality of life in MS caregivers and that male spousal caregivers had significantly higher scores than female spousal caregivers on the SF-36. Aymerich et al. [51] reported that while there were no significant differences by gender in physical HRQOL, women scored significantly lower on mental HRQOL than men MS caregivers [52]. Similar gender differences in HRQOL have been reported in caregivers of patients with other neurological conditions, such as Alzheimer's disease, amyotrophic lateral sclerosis, and stroke [53–55]. These gender differences may reflect how men and women vary in their perceptions of and coping with caregiving stress. It is possible that even though women experience and express greater disturbances in their health-related quality of life, their socially dictated need to continue their caregiving duties (despite the toll this may take on their personal well-being) makes them less likely to report that such disturbances limit their caregiving role. When the covariates were added to the model, the overall model was no longer statistically significant, suggesting that gender differences in these covariates may have accounted for some of the gender differences in HRQOL. However, it is important to note that the model approached statistical significance (*p* = 0.075), so follow-up ANCOVAs, if they had been performed, likely would have revealed some gender differences even after controlling for the covariates.

### 4.3. Gender Differences in Demographics

The analyses examining gender differences in demographics found that, in comparison to men, women caregivers had fewer years of education, were less likely to work outside the home, spent almost twice as many hours per week providing care (79.28 versus 48.48, *p* = 0.018), and had provided care for nearly three times as many months (66.31 versus 24.30, *p* = 0.002). These findings suggest a profound disproportionate burden of care in Latin America falling on women as opposed to men. This is consistent with the rigid gender roles in Latin America, which are likely influenced by a history of unequal distribution of resources within society. The vast majority of the population and families affected by serious illnesses (except for those in higher echelons) have access only to family care since it carries no monetary cost; this, in turn, may have the effect of reinforcing social specialization via traditional gender roles and assigning principal caregiving tasks to women [56]. Although gender differences in the relationship of caregivers to patients could not be taken into account in the MANCOVAs due to their multiple categorical nature, it is notable that 55.6% of men caregivers were the patient's spouse, while only 14.8% of women caregivers were. Conversely, only 11.1% of men caregivers were parents of the patient, whereas 63.0% of women caregivers were. It is unlikely that these demographic differences accounted for the gender differences in mental health and HRQOL found in this study because there was still adequate representation in the other relationship-to-patient categories, but it does beg for future studies to investigate why men MS caregivers in this region are more likely to be spouses and women MS caregivers are more likely to be parents. A potential reason could be parental caregivers being older on average than spousal caregivers and women simply having greater longevity than men, an epidemiological trend that might mean that there are more older mothers alive in families who are able to take on the caregiving role.

Within traditional Latin American cultures with strong gender-role divisions, women are expected to be dependent, chaste, and submissive, in contrast to the idealized masculine gender role of being independent, virile, and dominant [57]. While Latino cultures are not homogenous, rigid gender roles generally dictate that women play a central role in the organization and maintenance of family life and traditions [58]. From an early age, girls and women are socialized into their expected tasks of providing care for the family, while foregoing and sacrificing personal time, self-care, socialization, and employment opportunities for the well-being of children and husband providers, as well as helping maintain their families' social respectability [29, 59]. These social forces likely influence the significant gender differences in care provision variables observed in this study. In addition, women caregivers in the current sample scored lower on all scales of the SF-36 compared to a normative sample of Mexican women [60]. This suggests that an additional load of providing full-time care to a loved one with MS takes a significant toll on women caregivers' HRQOL, beyond that already experienced by Mexican women in the general population.

### 4.4. Clinical Implications

The findings from the current study have implications for MS caregiver interventions in Latin America and possibly for caregivers in other global regions or cultural groups with similar values and beliefs. MS caregiver interventions in Latin America—particularly for women caregivers—should address the influence of gender-role conformity on care and psychosocial functioning. While gender is not a variable that can be changed, the pervasive effect of roles expected of women in Latin America in the context of being the principal providers of care for individuals with MS is potentially malleable. For women MS caregivers, quality of life may be reduced because the time spent on caregiving tasks incurs an opportunity cost of giving up engaging in recreational, socialization, or self-care activities.

Mexican women, especially over the age of 40, have been reported to have a higher likelihood of engaging in poor self-care practices (e.g., lack of exercise, poor eating habits, poor sleep, obesity, and overweight), forego preventive medical procedures (e.g., breast self-exams or uterine and cervical cancer exams), only go to the doctor when ill, and lack social security [61], consistent with previously reported traditional expectations of self-sacrifice in the name of caring for one's family [29]. Clinicians can help women MS caregivers become aware of and perhaps renegotiate social roles which place an undue burden of care on them, a process that could improve women caregivers' mental health and HRQOL by reducing their caregiving load. Clinicians could also initiate discussions about the roles that women caregivers assume implicitly and increase caregivers' awareness of the negative physical and mental health consequences of neglected self-care. Clinicians could use the present findings to guide the assessment of caregivers' needs and plan their interventions accordingly. Family-based or systemic approaches may include strategies which lead to delegating care tasks, whenever possible and acceptable, to extended family members and other individuals who constitute the caregiver's social support group.

As was observed in this study's sample, women caregivers had fewer years of education and were less likely to work outside of home. It has been suggested that reducing work hours to accommodate the caretaking load may predispose caregivers to greater vulnerability later in life by accumulating fewer benefits such as social security and possibly by experiencing more poverty [62]. Creation and extension of social programs to assist family caregivers—especially women—such as respite or financial assistance also could have a positive effect on their mental health and HRQOL. As such services are very limited in Mexico and other Latin American countries [63], more funding and intervention at the policy level may be needed in order to establish the formal networks of support for informal caregivers, whose unpaid labor in Mexico accounts for up to 15% of the GDP [64].

### 4.5. Limitations and Future Directions

Despite the current study's implications for MS caregiver interventions in Latin America, it has several limitations and, as a result, directions for future research. First, the data are limited because they are cross-sectional and were only collected in one city in Mexico. The findings therefore do not take into account change over time, so trajectories of mental health, social support, and HRQOL cannot be examined. The findings also might not generalize to other countries in Latin America or Latinos in the US. Second, although gender-role conformity was extensively used as an explanation for the findings, no self-report measures of gender-role conformity were collected. This would be a ripe area for future research. Third, all participants volunteered to participate in the study, which does not exclude the possibility that these individuals presented with different levels of mental health, HRQOL, and social support than the majority of Mexican MS family caregivers who did not have access to the health facility from which caregivers were recruited. Fourth, perhaps the most significant limitation is that no measures of patient disability or needs were collected, so the potentially differential impact of disease symptoms on men and women caregivers' psychosocial functioning cannot be examined. A number of clinical variables should be assessed in future similar research including patient functional independence, MS symptoms, disability level, mobility and wheelchair needs, cognitive impairments, and secondary medical conditions. All of these variables can play a major role in caregiving and should be primary subjects of investigation in future research on gender differences in MS caregiver psychosocial functioning.

## 5. Conclusions

With these limitations in mind, this study represents the first to examine gender differences in mental health, HRQOL, and social support among MS caregivers in Latin America. The findings robustly suggest that, in Mexico, women assume substantially more caregiving duties, and therefore MS caregiver interventions in Latin America—particularly for women caregivers—should address the influence of gender-role conformity on care and psychosocial functioning.

## Figures and Tables

**Table 1:** Caregiver participant demographics.

Variable	Men (*n* = 27)	Women (*n* = 54)	*p* value
Age, years, mean (SD)	37.63 (15.65)	46.24 (14.46)	.016
Marital status %			NS
Single	33.3	31.5	
Partnered	66.6	68.5	
Relationship to patient %			.000
Spouse	55.6	14.8	
Parent	11.1	63.0	
Sibling	18.5	9.3	
Child	3.7	7.4	
Partner	11.1	0	
Professional caregiver	0	1.9	
Other	0	3.8	
Education, years, mean (SD)	13.41 (3.78)	10.91 (4.52)	.016
Working outside the home %	92.6	44.4	.000
Weekly time caregiving, hours, mean (SD)	48.48 (41.05)	79.28 (59.74)	.018
Total time caregiving, months, mean (SD)	24.30 (23.04)	66.31 (66.68)	.002

SD: standard deviation; NS: not significant.

**Table 2:** Mental health, social support, and health related quality of life scores for women and men.

Variables	Women	Men	*F*-statistic	*p* value	Cohen's *d *
Satisfaction with life	22.70 (6.27)	24.89 (6.39)	2.158	.146	.35
Depression	7.35 (5.48)	3.07 (3.85)	13.752	.000	.90
Burden	25.09 (16.61)	16.56 (7.78)	6.388	.014	.66
Social support: appraisal	10.83 (2.58)	13.30 (3.04)	14.593	.000	.88
Social support: belonging	11.50 (2.85)	13.40 (2.36)	9.013	.004	.73
Social support: tangible	10.81 (2.68)	13.15 (2.63)	13.836	.000	.88
Self-esteem	29.56 (5.52)	33.70 (4.61)	11.292	.001	.81
Anxiety: state	24.96 (12.17)	18.07 (9.77)	6.537	.012	.62
Anxiety: trait	27.63 (10.73)	18.31 (7.96)	15.469	.000	.99
Physical functioning	77.50 (24.57)	99.33 (10.65)	10.20	.002	.84
Role-physical	74.53 (35.85)	81.48 (25.56)	.81	.372	.22
Role-emotional	64.20 (41.39)	81.48 (32.47)	3.59	.062	.46
Vitality	51.48 (17.50)	71.11 (17.28)	22.83	.000	1.29
Emotional well-being	56.44 (19.34)	73.63 (17.72)	15.00	.000	.93
Social functioning	70.37 (21.21)	86.11 (14.84)	11.91	.001	.86
Pain	69.40 (21.98)	89.91 (15.14)	18.95	.000	1.09
General health	52.87 (19.54)	72.59 (15.65)	20.80	.000	1.11

*Note*. Cohen's *d* effect size: .20 = small, .50 = medium, and .80 = large. *p* values are two-tailed. Standard deviations are in parentheses.

**Table 3:** Covariate-adjusted mental health and social support scores for women and men.

Variables	Women	Men	*F*-statistic	*p* value	Cohen's *d *
Satisfaction with life	23.03 (6.80)	24.23 (7.15)	.46	.499	.17
Depression	6.76 (5.10)	4.25 (5.36)	3.60	.062	.48
Burden	23.35 (14.95)	19.99 (15.75)	.74	.393	.22
Social support: appraisal	10.95 (2.92)	13.07 (3.07)	7.90	.006	.71
Social support: belonging	11.81 (2.65)	12.78 (2.79)	1.99	.163	.36
Social support: tangible	10.93 (2.79)	12.92 (2.94)	7.53	.008	.69
Self-esteem	29.54 (5.65)	33.74 (5.94)	8.20	.005	.72
Anxiety: state	25.12 (12.21)	17.76 (12.84)	5.39	.023	.59
Anxiety: trait	27.78 (10.65)	18.01 (11.21)	12.22	.001	.89

*Note*. Cohen's *d* effect size: .20 = small, .50 = medium, and .80 = large. *p* values are two-tailed. Standard deviations are in parentheses.
